# Molecular dynamics simulations and docking enable to explore the biophysical factors controlling the yields of engineered nanobodies

**DOI:** 10.1038/srep34869

**Published:** 2016-10-10

**Authors:** Miguel A. Soler, Ario de Marco, Sara Fortuna

**Affiliations:** 1 MOlecular NAnotechnology for LIfe Science Applications (MoNaLiSA) Theory Group, Department of Medical and Biological Sciences, University of Udine, Piazzale Kolbe 4, 33100 Udine, Italy; 2 Center for biomedical sciences and engineering, University of Nova Gorica, Glavni Trg 8, 5271 Vipava, Slovenia

## Abstract

Nanobodies (VHHs) have proved to be valuable substitutes of conventional antibodies for molecular recognition. Their small size represents a precious advantage for rational mutagenesis based on modelling. Here we address the problem of predicting how *Camelidae* nanobody sequences can tolerate mutations by developing a simulation protocol based on all-atom molecular dynamics and whole-molecule docking. The method was tested on two sets of nanobodies characterized experimentally for their biophysical features. One set contained point mutations introduced to humanize a wild type sequence, in the second the CDRs were swapped between single-domain frameworks with *Camelidae* and human hallmarks. The method resulted in accurate scoring approaches to predict experimental yields and enabled to identify the structural modifications induced by mutations. This work is a promising tool for the *in silico* development of single-domain antibodies and opens the opportunity to customize single functional domains of larger macromolecules.

Nanobodies (VHHs) correspond to the heavy-chain variable region of camelid antibodies and are considered together with vNAR from sharks the smallest antibody fragments which still preserve the binding capacity of the whole original antibody they derive from^1^. They proved being valid substitutes of conventional IgG antibodies in basic research and diagnostics, and they are actively tested to confirm their therapeutic potential^2^,^3^,^4^. Their small mass (14–15 kDa, corresponding to 120–135 amino acids) has been considered an advantage for the preparation of high density antigen-capture surfaces in chromatography and microarrays, favors solid tumor penetration and simplifies *in vivo* imaging because of their rapid clearance enabling images acquisition already 1 hour after injection^2^. VHHs (as well as the human counterpart VH) can be easily engineered and produced inexpensively in microbial cell factories as fusion immune-reagents^5^,^6^. However, the variation of single amino acids can completely modify their structural and functional characteristics^7^,^8^, result in critical loss of stability^9^, and low or null yields of functional nanobodies^10^ due to aggregation.

VHH structural plasticity represents a still not thoroughly exploited but potentially invaluable advantage for their rational mutagenesis aimed at the identification of binders with improved characteristics specific for the different biotechnological applications in which these molecules can be used. The effect of both single mutations and grafting of larger regions (i.e., CDRs) has the potential of being evaluated without the need of time-expensive experimental validations. In fact, nanobodies can be modeled in shorter time, with greater accuracy and with substantially less computational resources than those required for IgG (1200 amino acids) but also for antibody fragments such as Fabs and scFvs. The possibility of developing rational optimization would be of enormous interest for instance in the case of antibodies with clinical potential, because usually their sequences must be humanized and improved for affinity but this process cannot be at the expense of stability and solubility^11^ to avoid loss of “usable yield”. In fact, even a contained yield variation might critically affect production costs. It would be therefore very profitable to identify an approach able to predict directly the effect of mutations on usable yields.

In general, individual protein biophysical features can be assessed by a number of computational approaches^12^, generally achieving the best accuracy with those more computationally expensive. This is why, following the progressive availability of computing power, the fastest sequence-based methods^13^ have been progressively replaced by structure-based protocols. The most popular being FoldX^14^,^15^, an empirical force field based program for the prediction of protein folding stability measured as *ΔG* variation, and CamSol^16^ and A3D^17^ which estimate the theoretical solubility *in vitro*. These computational approaches are very fast and can accurately predict mutants stability and solubility respectively. Their readouts suggest improved/altered biophysical behavior of protein constructs, which however may or may not result in better protein yields. Indeed, stability and solubility are not necessarily related to usable yield, and the available computational tools do not claim to correlate with higher protein yields. In fact it is known that there might even be no effect on yield upon improving solubility^18^. Further, it is worth noticing that the scoring evaluation methods used by these programs do not account for the long distance effects introduced by the mutation itself on the whole molecule structure nor on the possible domain-domain interactions typical of antibodies. These reasons might further explain why they are not directly applicable for estimating the VHH yields that mutants can achieve when produced as recombinant proteins. Reliable estimations of usable yields would rather require a modelling approach based on the accurate sampling of the antibody conformational space^19^ able to guarantee the simultaneous fulfilling of all the biophysical and biological requisites. In this respect, molecular dynamics (MD) simulations are one of the tools of choice in that they allow to explore thoroughly the conformational space of the system under investigation by following its time-evolution. Instead, the study of protein-protein interactions, especially when their interaction site is unknown, relies on docking. Docking algorithms allows pinpointing the reciprocal orientation of two macromolecules engaged in the formation of a dimer by exploring a number of poses and ranking them. A methodology taking advantage of a combination of the two will have the potential to address a wider range of problems. Since the computational cost increases rapidly with the antibody complexity, VHHs represent the simplest model to assess a new methodology. The literature relative to single-domain usable yields describes few critical factors: the VHH thermodynamic stability^18^,^20^,^21^, the propensity to misfold and/or aggregate because of inappropriate residues at specific positions^8^,^22^,^23^,^24^,^25^, and the colloidal instability, intended as the propensity of correctly folded monomers to form multimers that tend to precipitate^26^,^27^,^28^. Overall it emerges that VHH yields are the result of the complex interaction among several biophysical factors. Consequently, we addressed simultaneously these factors using an accurate and reliable *in silico* screening protocol for VHH usable yield prediction based on an innovative combined application of well-established modeling techniques: MD simulations and docking.

## Results

### Usable yields are not predicted with sufficient accuracy by rapid scoring-based programs

**Figure 1:**
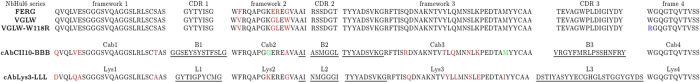
Representative sequences corresponding to the VHHs analyzed in this work. The first three illustrate the NbHul6 series. FERG-VGLW mutations are highlighted in red, while the W111R substitution is in blue. The last two are the wt sequences corresponding to the VHHs used for swapping. The fragments swapped between frameworks are underlined. In green are reported the discrepancies between literature and the PDB data.

We first assessed the thermodynamic stability and solubility of a number of VHHs, differing for either single point mutations (NbHul6 series)^10^ or swapped CDRs (chimera set)^29^, with publically available rapid scoring-based programs^14^,^16^,^17^. Residue numbers in NbHuL6 series followed those from the PDB file, in contrast to the experimental work^10^ in which residue numbers matched with the International ImMunoGeneTics information system amino acid numbering (imgt.cines.fr). The representative sequences of the nanobodies are reported in Fig. 1. Their experimental yields and *ΔG* variation (*ΔΔG*^exp^) with respect to the wild type (WT) have been published previously^10^,^29^ and are collected in Table 1. The NbHul6 series explores combinations of point mutations necessary to transform a VHH with the typical camelid FERG hallmarks in the framework 2 into its humanized version characterized by the VGLW signature, as well as the effect of the W111R point mutation in the framework 4 since it is apparently critical for the correct display of the CDR3 loop^10^,^30^. In this series the yield of most of the mutants is comparable to that of the WT with the exception of VGLG, VGLW, and FERG-R for which a much lower yield had been observed. The chimeras set was built by swapping the CDR loops between the unaltered frameworks of the VHHs cAbLys (Lys) and cAbCII10 (CII)^29^ with the effect of decreasing their yield with respect to the WT they originated from. NbHul6 mutants were all indicated as soluble, with the exception of VGLW which precipitated at 4 °C^10^, while no solubility information was provided for the chimeras set.

The computational output of selected scoring based methods (FoldX, CamSol, and A3D) was compared with the published experimental data. FoldX-calculated stability values of the NbHul6 series correlated with both the *ΔΔG*^exp^ and the VHH melting temperatures reported in the literature^10^ with the exception of the mutant VGLG (Table 1). Nevertheless, both experimental and calculated *ΔΔG* did not sufficiently correlate with the experimental yields, indicating that the variation of thermodynamic potential alone failed to predict correctly which mutations affected the usable yield. Both CamSol and A3D predicted the low solubility of the mutant VGLW (Table 1). Otherwise, the calculated solubility cannot be directly used as a predictor for usable yields: many scores did not correlate with the experimental yields (Table 1, in red), such as those attributed to FGLW, FGLG and VGLW (similar scores in both tests, significantly different yields) or FERG-R (good predicted solubility, poor usable yield).

In the case of the chimeras, the wild-type constructs Lys-LLL and CII-BBB were assumed as the references of each sub-group. In these sets, FoldX predictions did not correlate well with the experimentally determined *ΔΔG* (Table 1). In most cases, the solubility calculated by CamSol and A3D was not a reliable indicator of the experimental yields (Table 1).

Altogether, the data summarized in Table 1 show that usable yields cannot be predicted by measurement or calculation of single parameters such as free energy variation or solubility and underline that they are the result of a complex interplay of biophysical factors. Consequently, we addressed the problem with a more holistic approach based on molecular dynamics and docking.

### Thermodynamic and colloidal stability of NbHul6 VHH variants

**Figure 2:**
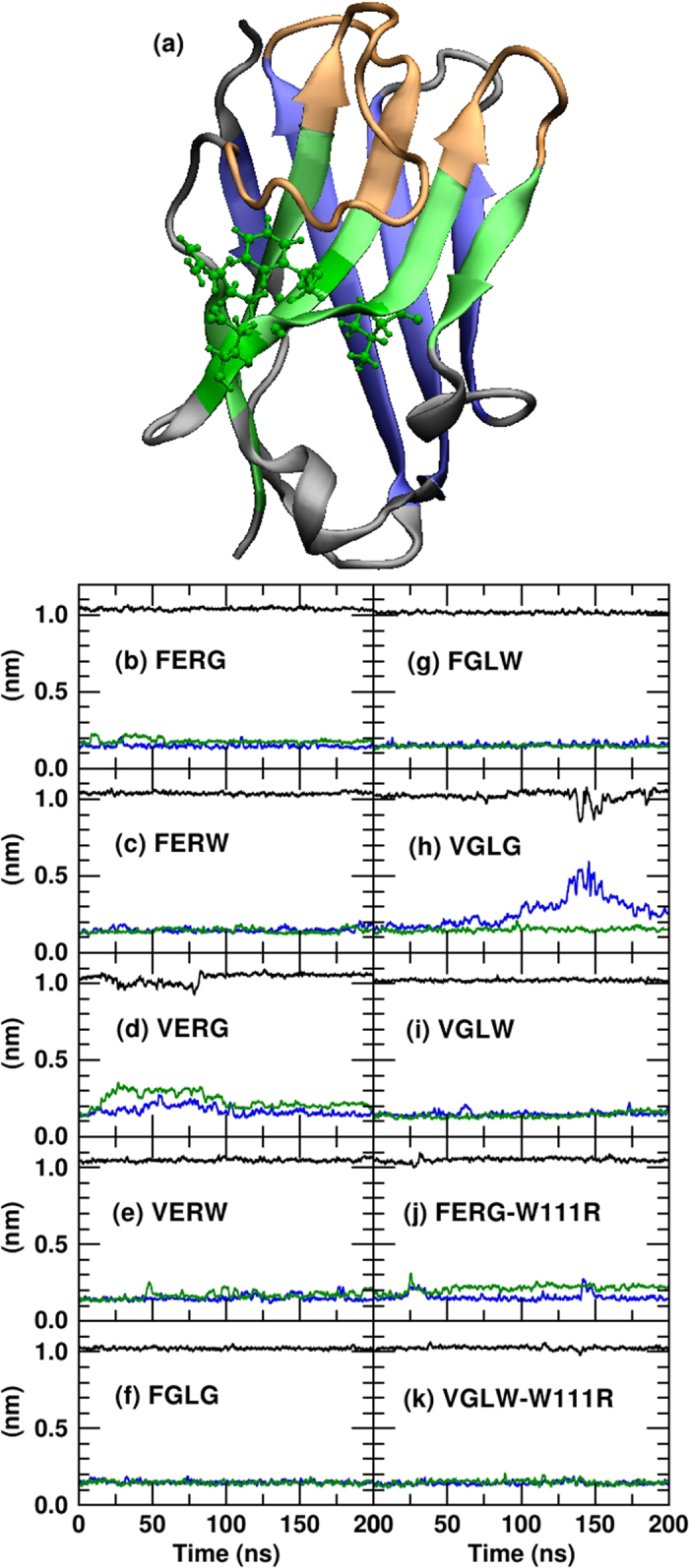
RMSD of NbHul6 mutants. (**a**) FERG-hallmark simulated representative conformation: the framework is composed by two sets of facing beta-sheets (green and blue), unstructured residues (gray), and three CDRs (orange). FERG fingerprint amino acids are as CPK representation. (**b**–**k**) RMSD of the two beta-sheet sets (green and blue) and distance between them (black) as a function of time at 500 K.

Molecular dynamics (MD) simulations (Suppl. Fig. S1a) at 300 K revealed that the point mutations did not lead to major structural rearrangements. Their Root Mean Square Deviation (RMSD) indicated only minor fluctuations. Backbone clustering was performed to identify representative conformations (see Methods and Suppl. Fig. S2). To evaluate their thermodynamic stability and identify the molecular regions more prone to unfolding, we run a set of MD simulations by heating each representative conformation at 500 K. We looked at the time development of the RMSD and of the center of mass distance of the facing pairs of beta-sheets (highlighted in blue and green in Fig. 2a). These order parameters enable to follow the unfolding process and in particular to see whether beta-sheets were lost (loss of secondary structure) or prone to diverge in a process leading to VHH “structural opening” (loss of tertiary structure^31^). Interestingly, the Val34 mutation seemed to be particularly critical for inducing long-distance structural perturbations since it affected the beta-sheets not directly interested by mutations, as illustrated by the fluctuations in the RMSD of the beta-sheets facing the mutated beta-sheet (Fig. 2d,e,h,i). The effect is more pronounced in VGLG (Fig. 2h) and VERG (Fig. 2d) whose beta-sheets distances along the 200 ns were also strongly affected. The simulations also indicated that the mutation G/W can compensate for the disruptive mutation Val34 and results in recovered stability of the beta-sheet structure (Fig. 2e,i). The same Trp44 mutation stabilized also the wild type FERG (Fig. 2b,c). More peculiar was the effect of the mutation W111R. It destabilized the other beta-sheets in FERG (Fig. 2j), but reduced the RMSD fluctuations in VGLW (Fig. 2k). FGLG, FGLW, and VGLW-R maintained their structure upon heating: their beta-sheet RMSD did not show any major fluctuation along the simulation time (Fig. 2f,g,k). The same is true also for FERW, VGLW, VERW.

These simulations predicted the experimental stability with relatively good accuracy in most of the cases (Table 1) but were still insufficient to explain the elevated usable yield of VERG and the low yields of VGLW. Since the analysis of long-distance structural perturbations due to single point mutations was inadequate to predict the experimental yield of the VHHs, we considered the additional contribution to aggregation due to the colloidal interactions among single molecules. This was studied by developing a semi-flexible docking based protocol.

**Figure 3:**
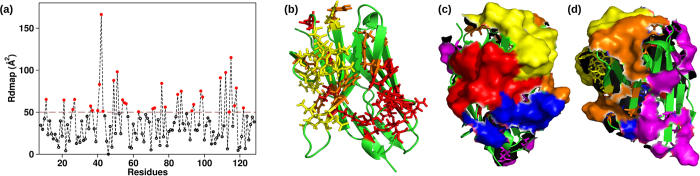
Identification of possible aggregation hotspots. (**a**) The global difference area contact per residue between the nanobodies VGLW and FGLW. (**b**) Residues identified by the InterProSurf tool^2^ (yellow) and by the difference contact maps (red) for the nanobody VGLW. Common identified residues are highlighted in orange. (**c**,**d**) Colloidal binding sites selected for nanobody VGLW are highlighted in different colors.

The docking results depend strongly on the defined active binding sites^32^,^33^,^34^. For this reason possible aggregation hotspots were pre-selected by applying a protocol that identifies binding sites according to the residue differences in contact maps between mutants and reference VHHs and to the analysis of solvent exposed hydrophobic regions^35^. For this analysis one representative conformation, the same formerly identified by backbone clustering, was chosen for each VHH. We used two complementary approaches to define specific surface regions as candidates to be aggregation hotspots. First, we focused on the structural differences between the mutated and the reference proteins by identifying the residues more affected by each mutation. We defined as reference VHHs the wild-type FERG and the mutant FGLW for the first VHH set (both have high yield and available crystal structures). We evaluated the residue contact area map of each VHHs by using the SPACE server^35^ without applying any threshold. We then calculated the difference between the contact area map of each mutant and that of the reference molecule averaged over the whole trajectory (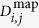, in Fig. S3) and evaluated the global difference contact area of each residue 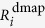 by summing up the absolute values of each column of the difference contact matrix:


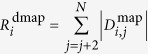


where for each residue *i* the summation is taken over all the residues *j* > *i + 1* up to *N*, the total number of residues. Residues with higher 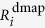 are those whose interaction with all the other residues changes more as an effect of the mutation. For each mutant we considered only those residues showing 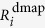 absolute values 20% above the average value (see Fig. 3a). An example of the output is shown in Fig. 3a where the mutant VGLW is evaluated with respect to the reference FGLW. The highest peak corresponded to the internal residue Arg35 next to the mutation Phe34 → Val. As a complementary approach, we analyzed each reference conformation with the web-tool protein-protein interaction predictor Interprosurf^32^ to identify the hydrophobic solvent-accessible residues, prone to interact with other protein groups. We then represented the residues selected from both approaches (see Fig. 3b) and we grouped those that shape binding surfaces and pockets defining possible aggregation hotspots (see Fig. 3c,d). The five larger sites were then chosen for further aggregation analysis.

Subsequently, the aggregation propensity of each single VHH was calculated by performing semi-flexible docking of all the possible interaction combinations of its monomeric structure. For each VHH, each identified aggregation hotspot was chosen in turn as the binding site to which the others were docked, obtaining 10 different possible dimers, with the exclusion of symmetric VHHs bound by means of the same site, as the VHHs here considered are known to be present as monomers in solution^9^. Since semi-flexible docking does not explore different backbone conformations of the protein, we used once again the most populated conformational state along the molecular dynamics trajectory formerly identified by clustering.

**Figure 4:**
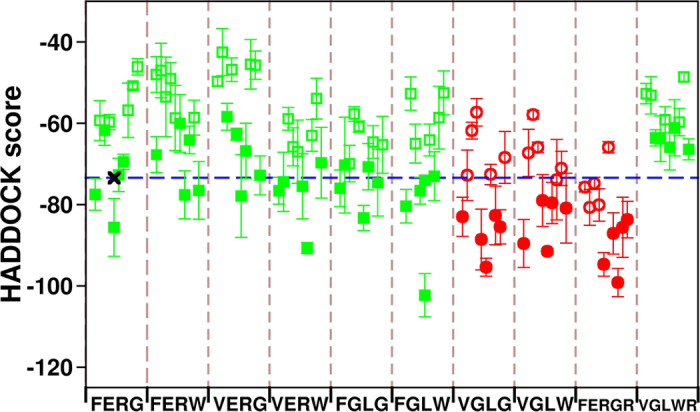
Docking score values obtained for the NbHul6 set of nanobodies. The scores are color coded according to the nanobody experimental yield: 3–4 mg/L (green squares), and 1–1.5 mg/L (red circles). Filled symbols indicate the five lowest values for each VHH. As a reference, the score corresponding to the average of the five lowest docking combinations of the wild-type FERG (black cross) is highlighted with a horizontal dashed line spanning all the results.

The docking results relative to the NbHul6 mutants (Fig. 4) and calculated with HADDOCK^33^,^36^ show dimers docking scores distributed between −40 and −110 for all the VHHs, with the most negative scores associated to strongly bound dimers. VHHs characterized by high experimental yields consistently scored less negative docking values (low aggregation propensity), whereas those with lower experimental yields scored more negative values (Fig. 4). This could be appreciated by looking at the five lowest score associated with each mutant consistently falling below the average score of the wild type VHH for all low yield VHH. Single outliers were identified, for instance in the case of FGLW, and could account for the low solubility predicted by the scoring-based methods (Table 1). Overall, this docking-based *in silico* approach allowed to discriminate the complete array of VHHs with similar yields from those difficult to express (VGLG, VGRW and FERG-R, Table 1).

### Thermodynamic and colloidal stability of VHH chimeras

**Figure 5:**
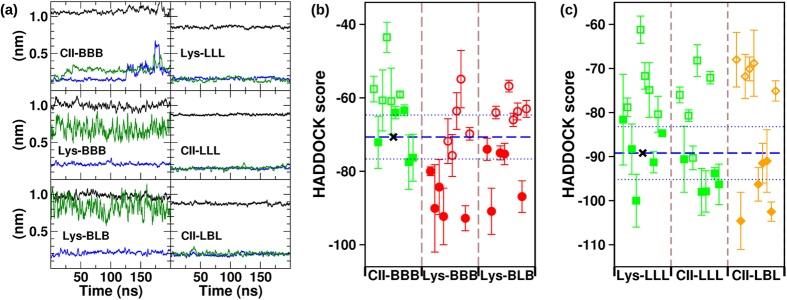
RMSD and docking score values obtained for the CII/Lys shuffled set of nanobodies. (**a**) RMSD of the beta-sheets sets (green and blue) and the distance between them (black) are reported as a function of time at 500 K. (**b**,**c**) The scores are color coded according to the nanobody experimental yield: 4–5 mg/L (green square), 2–3 (orange diamond) and 0–1 mg/L (red circle). Filled symbols indicate the five lowest values for each VHH. As a reference, the score corresponding to the average of the five lowest docking combinations of the wild-type CII-BBB and Lys-LLL (black crosses) are highlighted with a horizontal dashed line spanning all the results with standard deviation indicated by two dotted lines.

We further validate our protocol by assessing the usable yield of a second set of VHHs. Initially, molecular dynamics simulations of chimeras obtained by CDR swapping were run at 300 K. Structural rearrangements of the backbone were observed during the first 100 ns in all the clones (Suppl. Fig. S4a). Backbone clustering was performed leading to representative conformations and the conformational stability test was performed simulating chimeras heated at 500 K (Suppl. Fig. S4b). The RMSD of the beta-sheets as well as their distances are reported in Fig. 5a. In terms of RMSD, the grafting of the CII CDRs was not tolerated by the Lys framework that responded with large fluctuations. The reintroduction of one original CDR did not seem to stabilize the domain. In contrast, the RMSD results indicated that the CII framework can easily accommodate the Lys CDRs and a mixed combination of CDRs from both original scaffolds was tolerated as well. Both sets of results are consistent with the experimental *ΔΔG*^exp^ values, but did not predict the usable yields (Table 1). We applied also in this case the docking protocol to assess its capacity to better predict the colloidal stability of the different constructs. This analysis was restricted to molecules sharing the same number of residues, as the absolute docking scores depend on the molecular size. Under these conditions, it is possible to measure variations of chimeras with respect to a control of the same amino acid length and, indeed, the docking scores were consistent with the experimental yields of the grafted VHHs (Fig. 5b,c). In the case of the first subset (Fig. 5b), the chimera scores clustered towards values indicating higher binding propensity than wild type CII-BBB. Lys-BBB, which experimentally did not yield any soluble protein, was predicted to strongly aggregate as the result of the contribution of several independent association modes between monomers. In the case of Lys-BLB, the binding propensity between monomers remained elevated, but was less extreme than in the previous case, confirming the trend of the experimentally determined yields. The docking-calculated colloidal binding propensity of mutants originated by the grafting of Lys CDRs onto CII indicated a moderate decrease and this trend was more relevant for the construct with the retrofitting of one original CDR (Fig. 5c). Also in this case, there is full correspondence with the experimental data (Table 1). The predicted yields are even better defined by restricting the analysis to the binding combinations with the 5 lowest scores, as these are expected to be the most strongly involved in the aggregation process.

### Analysis of long distance effects of mutations

The exposed results show that the combination of MD and docking is an accurate predictor for usable yields of VHHs. Although computationally it is by far more demanding than conventional scoring approaches, in return it provides further relevant information for the identification –at the amino acid level resolution- of the molecular features involved in the aggregation process. We observed (Figs 4 and 5b,c) that the lowest scoring dimers for each of the VHHs, corresponding to the poorest experimental yields, consistently involved only one/two among the aggregation hotspots. This condition allows for further rationalization of the contrasting effect that single mutations can exert on the VHH yields and here we describe some paradigmatic examples.

**Figure 6:**
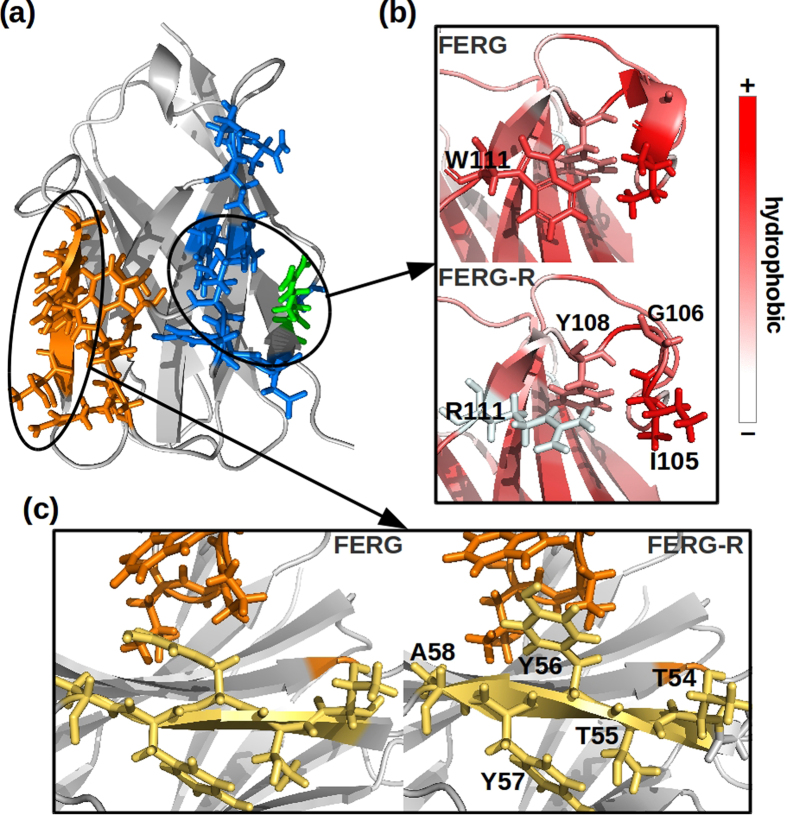
Long distance effect of a single mutation at the level of amino acid rearrangement. (**a**) Participating residues in the two aggregation hotspots of FERG-R (blue and orange, respectively) with highlighted mutated residue Arg111 (green), (**b**) close-up of contact differences and secondary structure changes between FERG-R and FERG in the first hotspot. Amino acids groups are color coded according to the Eisenberg hydrophobicity scale^50^, (**c**) structural details of the second hotspot.

In the case of the NbHul6 set, the mutation Trp111Arg in the FERG background (FERG to FERG-R) led to halving the experimental yield (Table 1). The two FERG-R aggregation hotspots responsible for the lowest binding scores are showed in Fig. 6a. Both are located in the framework-2/CDR3 region and in one of the cases the mutated residue participated actively in the binding. Observing the differences of solvent exposed residues in the first hotspot of FERG and FERG-R (Fig. 6b), the three CDR3 residues Ile105, Gly106, and Tyr108 become exposed only in FERG-R. Additionally, differences observed in the residue contact maps between FERG and FERG-R are mainly centered around the mutated residue, Arg/Trp111, and affected residues in both framework-2 and CDR3, such as Ile105. The bent structure of CDR3 present in FERG disappeared after the mutation. In the case of the second hotspot (Fig. 6c), the solvent exposed residues of the external β-sheet in framework-3, Thr55-Ala58, participated directly in the aggregation of FERG-R as a consequence of the torsion of the secondary structure upon mutation. Overall, while the substitution Trp to Arg was expected *a priori* to lead to a more soluble mutant due to the introduction of a positive charge at the domain surface, this docking analysis enabled to show that it also drove critical modifications in the native intradomain contacts. In particular, the rearrangement of the CDR3 loop and the framework-3 β-sheets resulted in the increase of stickiness between monomers.

**Figure 7:**
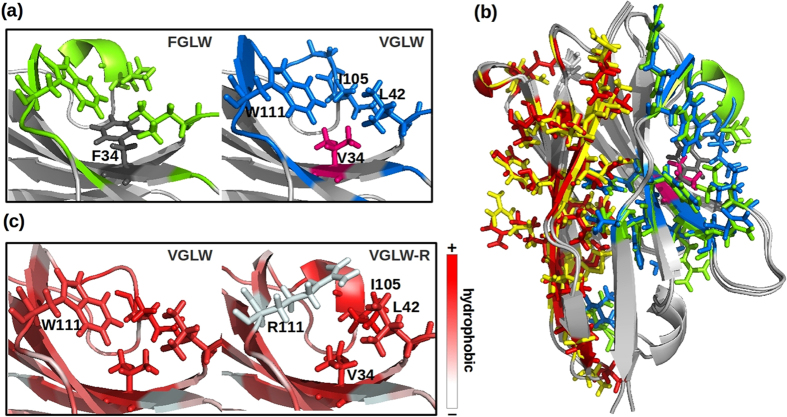
Molecular rearrangement induced by FGLW → VGLW → VGLW-R successive mutations. (**a**) Close-up of contact differences and secondary structure changes between FGLW and VGLW in the first hotspot, (**b**) structural comparison of both hotspots between FGLW (green and yellow) and VGLW (blue and red), and (**c**) close-up of contact differences and secondary structure changes between VGLW and VGLW-R where amino acids groups are color coded according to the Eisenberg hydrophobicity scale^50^.

The mutation Phe34Val (FGLW → VGLW) induced a yield decrease from 3–4 to 1 mg/L (Table 1). Surprisingly, neither Phe34 nor Val34 belong to aggregation hotspots since they are masked by other residues of the framework-2/CDR3 (Fig. 7a,b). This represents an interesting example in which the usable yield changes because of the mutation of a buried residue that exerted its effect by promoting the long-distance rearrangement of hotspot surface residues (Fig. 7a). The first VGLW hotspot was formed by residues belonging to framework-2/CDR3, while the second by a combination of residues of the frameworks 1 and 3 forming beta-sheets positioned opposite to Val34 (Fig. 7b). Both hotspots had residues whose side-chains are reoriented as an effect of the Val34 mutation with their consequent exposure to the solvent. These modifications are dramatic in the case of the residues present in the first hotspot, where the CDR3 loop lost its bent structure (Fig. 7a).

The mutation Trp111Arg in VGLW (VGLW → VGLW-R) affected positively the usable yield, in contrast to the former two described mutations (Table 1). In this case, the mutation led to the rearrangement of the CDR3 and the framework-4 and to the reorganization of single amino acid side-chain exposure to the solvent (Fig. 7c). Specifically, the Arg111 side-chain masked the hydrophobic groups of Val34 and Leu42 extremely more efficiently than Trp111, which contributed to form a sticky spot prone to aggregate. As a consequence, residues of CDR3 and of the framework-4 relocated their backbones involved in the stabilization of the framework-2 hydrophobic surface. In this example, the CDR3 assumed a bent structure after the mutation that was not present in VGLW, suggesting that its spatial orientation plays a direct role in the VHH colloidal stability.

Among the low yield chimeras, the Lys-BBB had three equally favorable aggregation hotspots instead of two, as formerly observed for the VHHs of the NbHul6 series. The structural comparison between the wild-type CII and the grafted Lys-BBB (Suppl. Fig. S5) evidenced more pronounced backbone differences than in the case of VHHs presenting point mutations preventing a detailed comparative analysis. These large-scale rearrangements affected directly the residues of all three hotspots, since the CDR3 region participates in all of them and the CDR2 loop in the second.

## Discussion and Conclusions

So far, the attempts to identify instability/aggregation hotspots in the VHH sequence by using either statistic approaches or static structural models that do not consider the dynamic spatial modifications induced by mutations failed to produce convergent and generally applicable results^8^,^18^,^22^,^23^,^24^,^37^. Consequently, also the strategies to improve the VHH stability remain case-specific and request empirical attempts to identify the necessary *ad hoc* adjustments^37^,^38^,^39^. The holistic modeling approach based on molecular dynamics simulations coupled with docking illustrated in this work allows taking into account the complexity of inter- and intra- molecular interactions expressed by VHH. Such complexity, extremely elevated even in these small single-domain structures, impairs empirical methods to be applied systematically. Specifically, we have proposed a new application of the already widely used HADDOCK program to obtain both usable yield predictions and information relative to the residues able to influence the domain stability. This protocol succeeded in predicting the experimental usable yields of engineered nanobodies with higher accuracy with respect to scoring methods evaluating single biophysical features of isolated molecules without taking into account dimer interactions. In particular, it showed that usable yield is influenced more by colloidal interactions than by the absolute solubility of single domains.

The docking results, function of aggregation hotspots, are interpreted in terms of usable yield by evaluating two key aspects: the number of combinations leading to dimers (aggregation seeds) and the strength of such binding events. This analysis was performed considering the surface hotspots potentially available for dimerization. Higher is the number of aggregation hotspots enabling high-affinity bonds, higher is the probability that such stickiness occurs at equilibrium leading to multimerization and precipitation. We also considered simplifying the analysis by restricting it to the five lowest scores for each VHH. These represent the most likely dimeric candidates of each set and consequently are responsible for most of the aggregation seeds. Although the proposed analysis does not consider the biological processes involved in recombinant antibody production (chaperoning, degradation, secretion efficiency) and that the aggregation seeds could modify the dynamic of colloid clustering over the time, it shows that the distribution of HADDOCK “dimerization” values is already a robust descriptor able to capture and predict the trend of usable yields in both nanobody sets.

The proposed approach was also able to characterize the spatial behavior of single residues as a function of mutations and, notably, to explain how these could induce long-distance rearrangements. The nature of the interaction between monomers was analyzed by using backwards the protocol and involved: (i) the identification of the colloidal hotspots responsible for the lowest scoring dimers obtained by docking, and (ii) the analysis of the hotspots residues rearranged after the mutation. The latter, predicted to influence the protein-protein interaction, were identified through their difference contact area maps and hydrophobic interface analysis. Our analyses showed that a single mutation can affect the aggregation propensity of the nearby region of the mutated residue, but also distant regions in the protein due to a domino effect of residue-residue interactions. For this reason, even though the mutated residue is not surface exposed, as in the case of the Phe34 → Val mutation, its mediated effect led to major deviations in the usable yield by modifying the solvent exposure of non-contiguous surface residues. Likewise, the different residue-residue interactions and the residue backbone rearrangements in CDR3 after the mutation Trp111 → Arg in FERG and VGLW explain the opposite response in terms of usable yield of such mutants. These conclusions imply that successful nanobody engineering should consider the overall effect of (single) mutations on the single-domain and how these influence the binding to other independent domains. Long distance effects on thermodynamic stability, which are difficult to be accounted for with scoring based methods, emerge clearly during molecular dynamics simulations. Extreme is the case of CDR grafting, which results in strong fluctuations of the framework leading in turn to a loss of structural stability and to the exposure of molecular surfaces with modified aggregation propensity.

These evidences complete the information from previous experimental works (reviewed in ref.^38^) which already demonstrated the participation of CDRs in the aggregation hotspots and confirm that minimal differences in these sequences could result into dramatic deterioration of VHH biophysical properties. The direct consequence is that the identification of a universal scaffold in which to accommodate any CDR without losing functionality and/or stability of part of the clones is challenging, if not merely improbable. This does not mean that synthetic single-domain antibody libraries built on a unique framework cannot be conceived to isolate effective binders^22^,^25^,^29^,^40^, but rather that their total diversity randomly introduced as hyper-mutated CDRs probably does not automatically result in a quantitatively corresponding functional diversity^7^. The diversity loss due to aggregation of a consistent part of the clones might have contributed to the poor performance of some synthetic libraries in comparison to pre-immune naive and immune libraries of theoretical smaller diversity^41^,^42^ but the binders of which have passed the *in vivo* quality control and consequently are all functional.

The encouraging take-home message is that the proposed method gives accurate results which out-perform the faster scoring-based ones by taking explicitly into account long-distance effects due to mutations and CDR grafting. On the other hand, its more detailed analysis is more computationally demanding. We have found that when different scoring-based programs (FoldX, Cam-Sol and A3D) provide convergent results, these can be predictive of the effect of single point mutations on usable yields. This approach becomes less reliable when more complex modifications are introduced, as in the case of chimeras.

At the present, modeling a VHH following the method exposed in this work and using a state-of-the-art machine is already at least as fast as *in vitro* screening methods used for identifying mutants with improved stability^43^. Considering the fast increase in computing power in comparison to the possibility to enhance the high-throughput approaches in wet-lab, it seems meaningful to invest in a computational method with the potential to be applied as it is to a variety of systems of biological interest such as enzymes and independent domains of larger proteins.

## Methods

### Homology Modeling

We used the 3EBA^10^ framework for the NbHuL6 series, the 1JTP^44^ and 1ZMY^29^ for the cAbLys and cAbCII10 frameworks respectively. The B loops were taken from 3DWT NbBCII10 and the L loops from 1ZMY (as indicated in Fig. 1). Glu29 e Tyr30 were missing in B1 and have been added with Swiss-PdbViewer 4.1^45^. We found some discrepancies between the PBS and the sequences reported in the literature in CII3: residue 93 (in literature there is Ile while in 1ZMY it is Met), while in CII2 there is no Gln between Gly43 and Glu44. The structures were aligned and the chimeras built manually from the aligned structures. The mutations were done with Swiss-PdbViewer.

### Stability and solubility calculations

We calculated FoldX stability values following the recommended protocol from the authors^15^. Thus, we used first the RepairPDB command to correct the homology modeling structure of wild type VHHs. Second, we evaluated the stability of different mutations employing the PositionScan command. We looked at scores relatives to the wild type. We defined 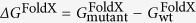 where 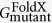 is the mutant FoldX score and 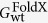 that of the wild type. Positive 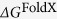 values identify mutants more thermodynamically stable than the wild type.

We calculate the CamSol solubility using the webserver tool of each homology modeling structure, and applying the cut-off (+/−0.7) summation of all residue scores as authors recommended^16^. Furthermore, we evaluate the A3D solubility of the homologous structures using the webserver tool with the Dynamic mode activated and a distance of aggregation analysis of 10 Å, according to the authors recommendation^17^. About the predicted solubility, 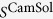 is inversely related to the solubility, while the magnitude of 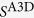 goes with the mutant solubility.

### Molecular Dynamics Simulations

We minimized the free molecules, placed the molecules in a cubic box with a water layer of 0.7 nm and performed a second minimization. We used AMBER99SB-ILDN^46^ force field and Simple Point Charge water before performing NVP and NPT equilibrations for 100 ps, followed by 200 ns NPT production run at 300 K. NVT simulations at 500 K have been run for selected configurations. The iteration time step was set to 2 fs with the Verlet integrator and LINCS^47^ constraint. We used periodic boundary conditions. All the simulations and their analysis were run as implemented in the GROMACS package^48^. RMSDs have been plotted as running averages over 100 sampled points. Simulations were run on MareNostrum III (BSC-CNS, Spain) and Galileo (CINECA, Italy).

### Clustering

We clustered 3800 structures extracted from the last 180 ns of the molecular dynamics simulations using the g_cluster program of GROMACS^48^. We employed the gromos method^49^ for clustering the structures, selecting as RMSD cut-off value the RMSD average value obtained from the matrix of structure combinations. Clusters with less than 100 structures were not considered. We considered only the backbone structure of the most fluctuating regions of the protein by selecting residues whose C-alphas RMSF values were above their average value, avoiding the extremes of the protein (see Suppl. Fig. S2). For each VHH, the cluster analysis led to 3–5 clusters with the most populated one accounting for the 80–90% of the sampled configurations. One reference conformation for each VHH was then chosen for further analysis.

### Docking

We used the web interface of HADDOCK^36^ with its standard parameters. System dependent active residues were defined for each nanobody using the protocol explained in Suppl. Information, while passive residues were automatically defined. Among the docking results obtained, we chose the docking cluster with the lowest score and containing more than 10% of total binding structures (around 150–200 docking structures attempted). We observed that clusters beyond this size had higher score standard deviations than our comparative range.

## Additional Information

**How to cite this article**: Soler, M. A. *et al*. Molecular dynamics simulations and docking enable to explore the biophysical factors controlling the yields of engineered nanobodies. *Sci. Rep*. **6**, 34869; doi: 10.1038/srep34869 (2016).

## Supplementary Material

Supplementary Information

## Figures and Tables

**Table 1:** Solubility and stability experimental and computational data of the mutants and chimeras explored in this work.

Name	Experimental Data		Calculated values		
	Yield (*mg/L*)	*ΔΔG*^exp^ (*kcal/mol*)	*ΔΔG*^FoldX^ (*kcal/mol*)	*S*^CamSol^	*S*^A3D^
**FERG**	**3.0**	**0.0**	**0.0**	**0.0**	**−71.3**
FERW	3–4	−2.5	−2.6	−2.0	−74.4
VERG	3–4	1.0	2.7	0.1	−74.3
VERW	3–4	−2.2	−0.2	−2.1	−72.1
FGLG	3–4	−5.6	−2.1	−11.5^#^	−61.7^#^
FGLW	3–4	−3.3	−4.2	−11.3^#^	−59.8^#^
VGLG	1.4	−4.0	0.7^#^	−17.3	−47.0
VGLW*	1.0	−3.0	−2.0	−12.4*	−59.1*
FERG-R	1.5	6.1	2.8	3.9^#^	−65.3^#^
VGLW-R	3.0	−1.2	−1.3	−6.9^#^	−61.1^#^
**CII**	**5.0**	**0.0**	**0.0**	**1.7**	−**76.0**
Lys-BBB	0.0	−	5.8	−2.5^#^	−88.1^#^
Lys-BLB	1.0	8.1	9.8	−0.3^#^	−97.1^#^
**Lys**	**5.0**	**0.0**	**0.0**	−**17.5^#^**	−**71.0^#^**
CII-LLL	4.2	−3.3	4.0^#^	−19.2^#^	−58.7
CII-LBL	2.5	0.0	12.1^#^	−18.8	−60.0

The first antibody set refers to the mutants described in ref. 10 and the indicated residues (nanobody hallmarks) are: 34F, 41E, 42R, 44G, 111W (Fig. 1). The second nanobody group is described in ref. 29. The reference (wt) VHHs for each group are in bold. *∆G*^FoldX^, *S*^CamSol^, *S*^A3D^ have been calculated as described in the text (^#^) Discrepancies with experimental data.

(*) Precipitates at 4 °C^10^.
