# Supplementary material for: Molecular dynamics simulations and docking enable to explore the biophysical factors controlling the yields of engineered nanobodies
Source: Sci Rep. 2016 Oct 10;6:34869. doi: 10.1038/srep34869 (PMC5056509; doi:10.1038/srep34869)
Supplement: Supplementary Information [file srep34869-s1.doc]

**Molecular dynamics simulations and docking enable to explore the biophysical factors controlling the yields of engineered nanobodies**

**Miguel A. Soler^1,#^, Ario de Marco^2,+^, Sara Fortuna^1,2^,*^,$^**

^1^MOlecular NAnotechnology for LIfe Science Applications (MoNaLiSA) Theory Group, Department of Medical and Biological Sciences, University of Udine, Piazzale Kolbe 4, 33100 Udine, Italy

^2^ Center for biomedical sciences and engineering, University of Nova Gorica, Glavni Trg 8, 5271 Vipava, Slovenia

e-mail: *sara.fortuna@ung.si ^$^web: www.sarafortuna.eu

e-mail: ^#^MiguelAngel.Solerbastida@uniud.it

e-mail: ^+^Ario.deMarco@ung.si

**I. SUPPLEMENTARY FIGURES.**


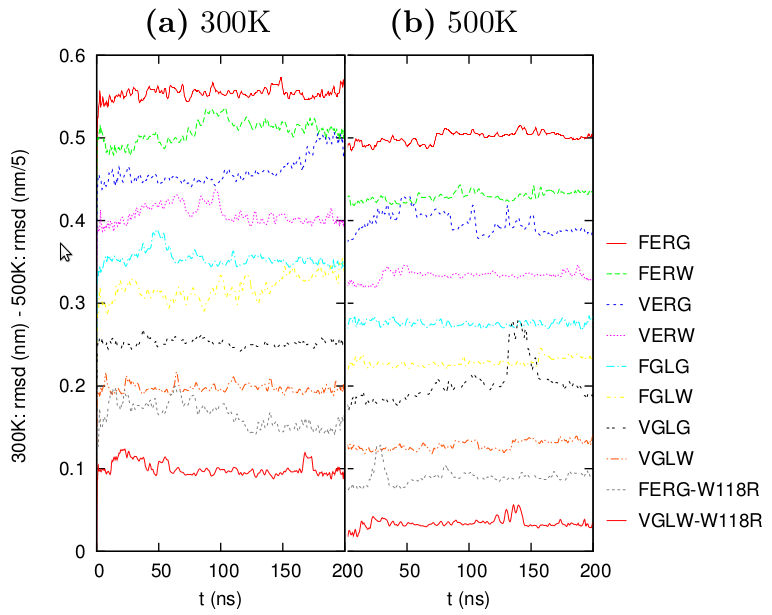


**Figure S1.** Backbone RMSD along MD trajectories for the NbHul6 series simulated at (a) 300 K and (b) 500 K (the latter rescaled by a factor of 5). Curves are displaced by 0.05 nm and smoothed with a Bezier curve over the 20,000 sampled configurations


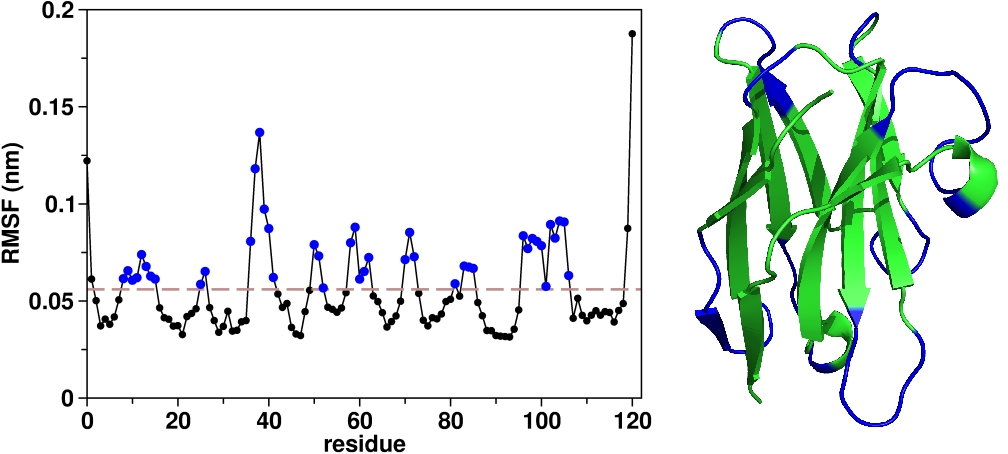


**Figure S2.** RMSF values of C-alpha atoms of nanobody FERG (left) and its 3D representation with the fluctuating regions highlighted in blue (right).


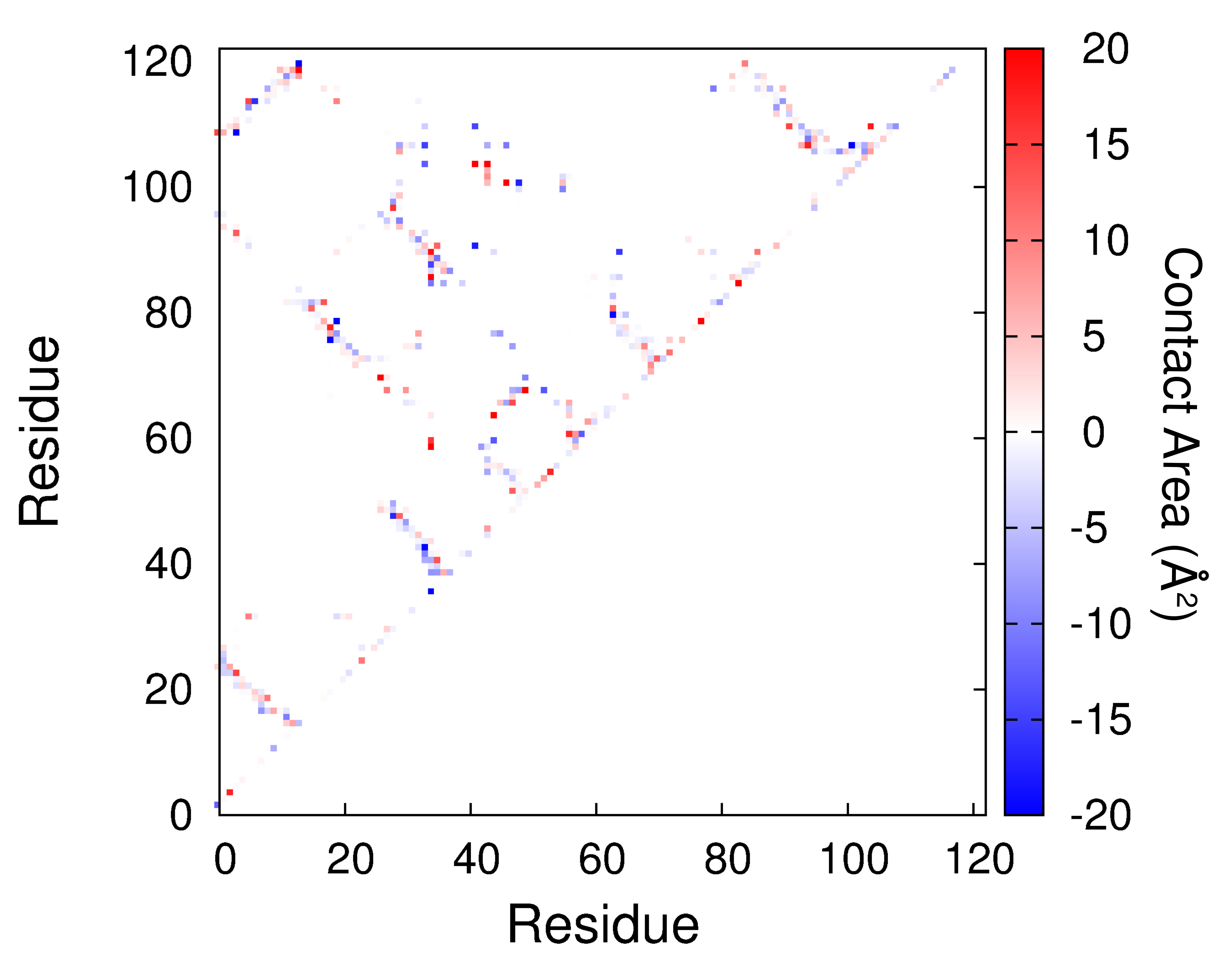


**Figure S3.** Difference residue area contact map.


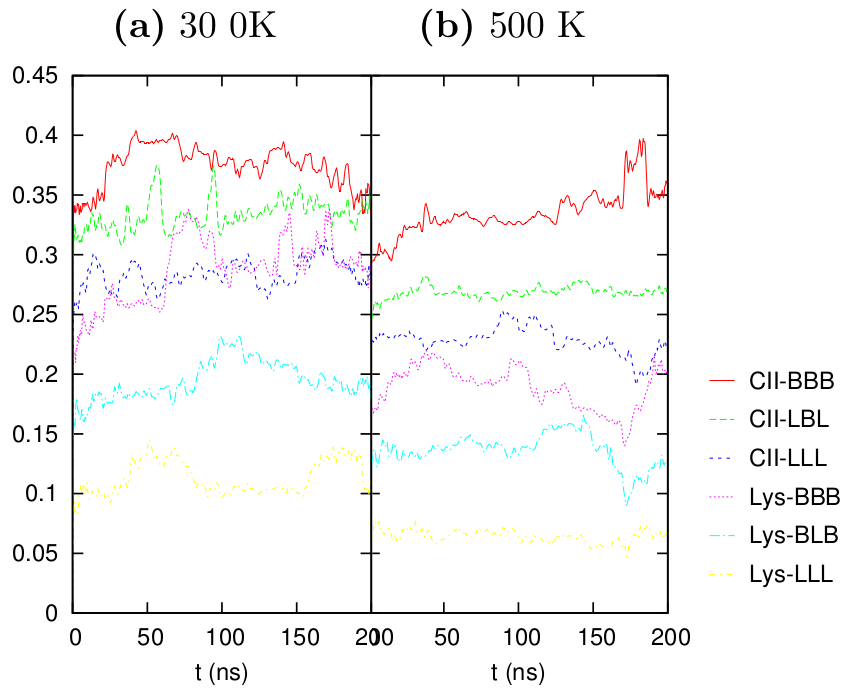


**Figure S4.** Backbone RMSD along MD trajectories for the CII/Lys chimeras at (a) 300 K and (b) 500 K (the latter rescaled by a factor of 5). Curves are displaced by 0.05 nm and smoothed with a Bezier curve over the 20,000 sampled configurations


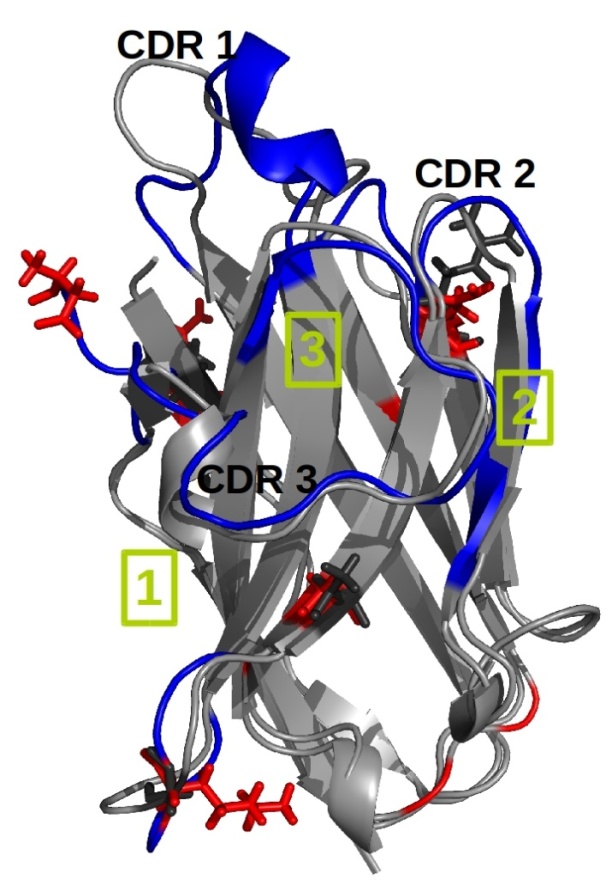


**Figure S5.** Structural alignment of CII and Lys-BBB. The backbone differences between proteins are highlighted in blue, while the different residues between frameworks CII and Lys are showed in red. Green numbers indicate the three aggregation hotspot regions found in Lys-BBB.

**II. SUPPLEMENTARY DISCUSSION.**

**A. RMSD at 500K.**

The RMSD values at 500K in the NbHul6 set (Fig. S1b) showed that, while larger RMSD fluctuations are expected (and their values are rescaled by a factor 5 in Fig. S1b), their mean values remained constant along the simulation time for most systems. Exceptions are VERG, VGLG, and FERG-R all expressing major rearrangements in the form of a large smooth increase (up to 0.25 nm) at the beginning of the simulation followed by fluctuations for VERG, and localized peaks as high as 0.25 nm for the other systems. FERG ad VERW showed minor rearrangements at 75 and 40 ns, respectively.

For the chimeras set, large RMSD fluctuations were observed for CII-BBB and Lys-BBB (Fig. S4b) in the form of localized peaks as high as 0.25 nm for CII-BBB and a large smooth increase (up to 0.25 nm) at the beginning of the simulation followed by fluctuations only in the case of Lys-BBB.
